# Cooperative Binding and Stepwise Encapsulation of Drug Molecules by Sulfonylcalixarene-Based Metal-Organic Supercontainers

**DOI:** 10.3390/molecules25112656

**Published:** 2020-06-08

**Authors:** Tian-Pu Sheng, Xin-Xia Fan, Guo-Zong Zheng, Feng-Rong Dai, Zhong-Ning Chen

**Affiliations:** 1 State Key Laboratory of Structural Chemistry, Fujian Institute of Research on the Structure of Matter, Chinese Academy of Sciences, Fuzhou 350002, Fujian, China; shengtianpu@fjirsm.ac.cn (T.-P.S.); czngroup@fjirsm.ac.cn (X.-X.F.); Zhengguozong@fjirsm.ac.cn (G.-Z.Z.); 2 University of Chinese Academy of Sciences, Beijing 100049, China

**Keywords:** host–guest chemistry, binding cooperativity, stepwise encapsulation, Hill equation, drug delivery

## Abstract

The cooperative binding behavior of a face-directed octahedral metal-organic supercontainer featuring one *endo* cavity and six *exo* cavities was thoroughly examined in chloroform solution through ultraviolet-visible (UV-Vis) titration technique using two representative drug molecules as the guests. The titration curves and their nonlinear fit to Hill equation strongly suggest the efficient encapsulation of the guest molecules by the synthetic host, which exhibit interesting cooperative and stepwise binding behavior. Based on the control experiments using tetranuclear complex as a reference, it is clear that two equivalents of the guest molecules are initially encapsulated inside the *endo* cavity, followed by the trapping of six additional equivalents of the drug molecules through six *exo* cavities (1 eq. per *exo* cavity), and the remaining guests are entrapped by the external pockets. The results provide an in-depth understanding of the cooperative binding behavior of metal-organic supercontainers, which opens up new opportunities for designing synthetic receptors for truly biomimetic functional applications.

## 1. Introduction

Binding cooperativity effects are a well-documented phenomenon and an essential principle prevalent in the fields of biology and supramolecular chemistry [1,2,3,4,5,6,7]. Well-known examples include allosteric binding of oxygen by hemoglobin in biology and metal ions chelation process by ethylenediaminetetraacetic acid (EDTA) in chemistry. Cooperative binding has been proven to be a very important mechanism that regulates molecular recognition behavior [2,3] and self-assembly processes [4,8,9] in biology and supramolecular chemistry. To date, a cooperative effect has been widely explored in biologic systems, and different basic principles have been developed to understand a wide range of biological phenomena [2]. However, cooperative modulation of macromolecular assemblies and guest binding behaviors in synthetic supramolecular systems remains poorly illustrated and understood.

Over the years, considerable attention has been paid to coordination containers possessing a well-defined hollow space that is suitable for guest encapsulation and allows them to be widely applied in a number of applications, such as guest recognition [10,11,12], drug delivery [13,14], and supramolecular catalysis [15,16]. A new class of coordination containers, namely, metal-organic supercontainers (MOSCs), incorporating macrocyclic container thia- or sulfonyl- calix[4]arenes as precursors [17], have been well demonstrated by us and others [18,19,20,21,22,23,24,25,26,27,28]. The MOSCs are demonstrated to possess several unique features such as their modular synthesis, robust architecture, and multiple binding domains (containing both *endo* and *exo* cavities). The unique multicavity structures allow MOSCs to be promising in various applications including guest regulation [21,22,23] and electrocatalysis [24,29]. Although encapsulation of multiple guests by MOSCs through their multiple binding domains has been demonstrated [20], the exact mechanism of binding cooperativity and/or binding regulation of those synthetic receptors remains unclear.

In the present study, we report a systematic study of the cooperative binding behavior of a biomimetic MOSC by using the two well-known gastric proton pump inhibitors, (*R*)-(+)-rabeprazole sodium and (*S*)-(−)-pantoprazole sodium, as representative guests. The cooperative binding profiles of the guests by the MOSC were carefully examined through ultraviolet-visible (UV-Vis) spectroscopic titration technique, which clearly revealed that stepwise encapsulation of the guests occurred sequentially, first within the *endo* cavity, then the *exo* cavities, and finally the external pockets of the MOSC. Each of these steps featured different degrees of binding affinity and cooperativity. Our results thus illustrate a rare example of stepwise, cooperative binding, and provide an in-depth understanding of such unique binding properties of a biomimetic receptor molecule that features multiple binding domains.

## 2. Results and Discussion

**Scheme 1:**
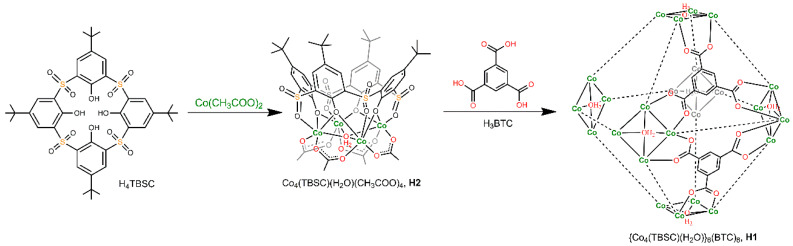
The self-assembly of host molecules **H1**^18^ and **H2**^30^ from *p-tert*-butylsulfonylcalix[4]arene (H_4_TBSC); the TBSC units in **H1** molecule are omitted for clarity.

**Figure 1:**
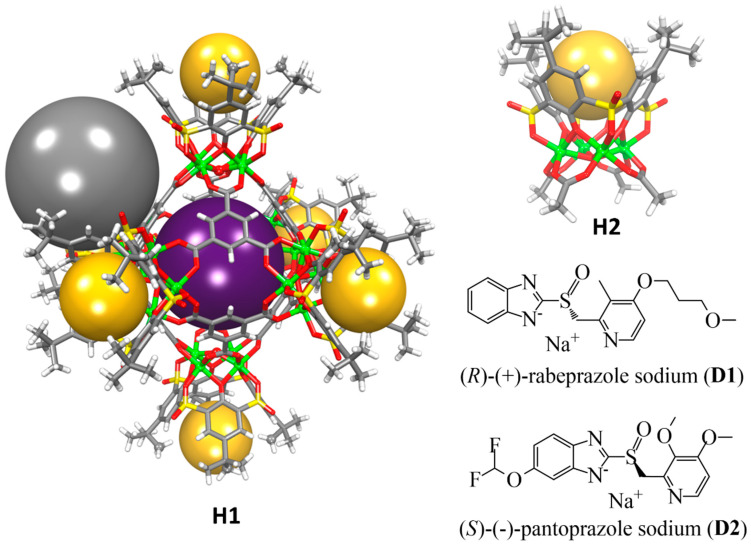
The crystal structures of host molecules (**H1** and **H2**) based on the CIF files reported in the original literature [18,30] and chemical structure diagrams of guest molecules (**D1** and **D2**) used in this study. The spheres serve to guide the eyes representing the *endo* cavity (purple), six *exo* cavities (yellow), and one of the eight external pockets (gray).

The metal-organic supercontainer MOSC-1-Co^18^, labelled here as **H1**, is constructed from six tetranuclear complex units [30] connected by eight units of the 1,3,5-benzenetricarboxylate (BTC) linker (Scheme 1). It features a dual-pore architecture containing two types of well-defined cavities: an *endo* cavity (with an inner diameter of ca. 1.4 nm and an estimated internal volume of 0.55 nm^3^) and six open-ended *exo* cavities (with an opening of ca. 0.8 nm × 0.8 nm and a depth of ca. 0.7 nm) (Figure 1). There are also eight external pockets defined by the BTC linker and three adjacent TBSC units. The multiple binding domains make the **H1** an intriguing and effective synthetic receptor for investigating efficient guest regulation and/or encapsulation, as well as binding cooperativity. To elucidate the guest binding behavior of supercontainer **H1**, the host–guest interaction profiles in solutions were carefully examined using the UV-Vis titration technique [31].^31^ Two drug molecules, namely, (*R*)-(+)-rabeprazole sodium (**D1**) and (*S*)-(−)-pantoprazole sodium (**D2**) (Figure 1), were selected as the guests. The synthetic precursor of **H1**, that is, a tetranuclear complex, labelled as **H2**, which mimics the structure of the *exo* cavities of **H1**, was chosen as a control host molecule to simulate the binding behavior of the *exo* cavities of the **H1**.

**Figure 2:**
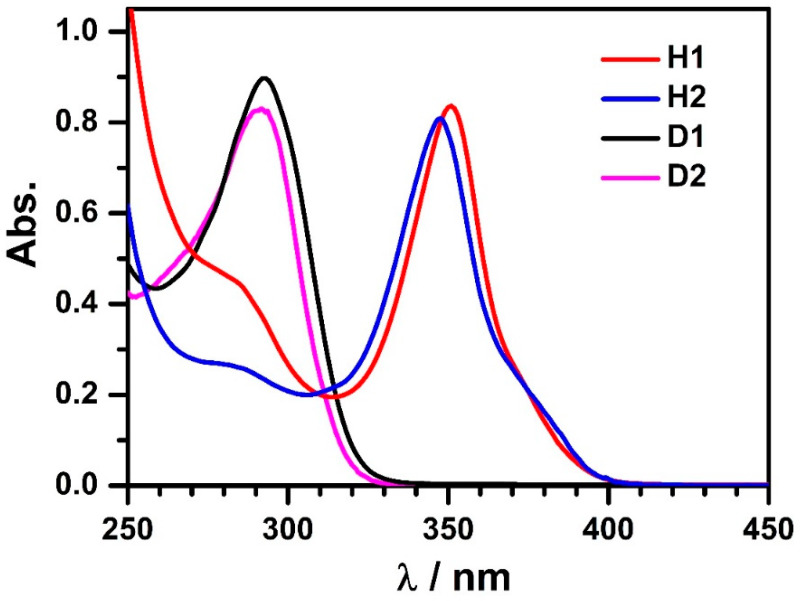
The ultraviolet-visible (UV-Vis) spectra of free hosts (**H1** and **H2**) and guests (**D1** and **D2**) in chloroform solution.

**Figure 3:**
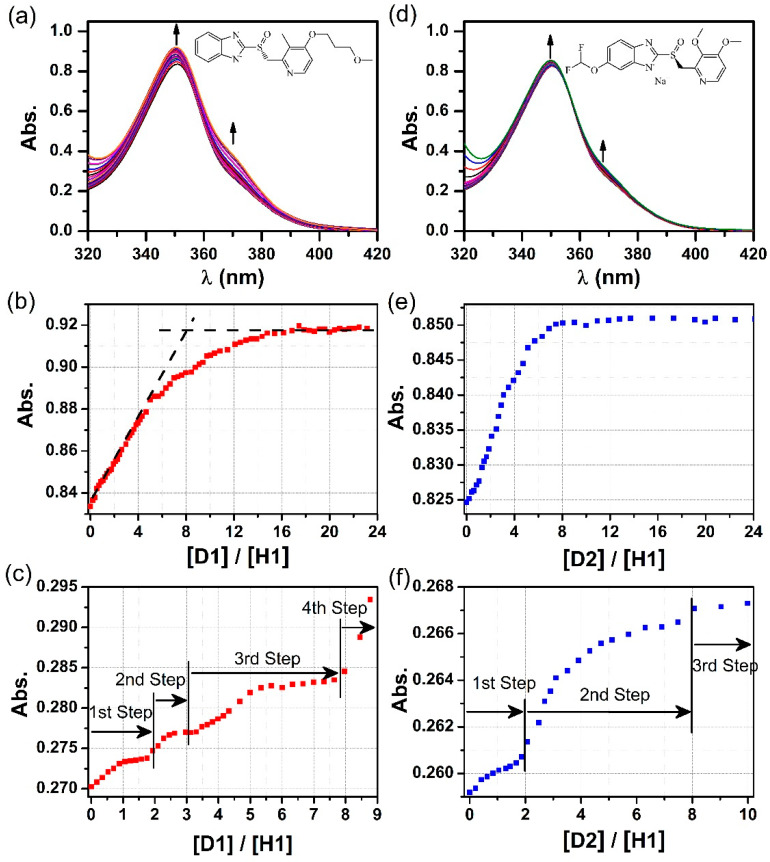
(**a**) UV-Vis spectra of host **H1** upon titration with **D1**, and plots of **D1**/**H1** molar ratio vs. absorbance at (**b**) 350 nm and (**c**) 370 nm based on the titration experiment. (**d**) UV-Vis spectra of host **H1** upon titration with **D2**, and plots of **D2**/**H1** molar ratio vs. absorbance at (**e**) 350 nm and (**f**) 370 nm based on the titration experiment.

The absorption spectrum of **H1** in CHCl_3_ features an intense and characteristic absorption band centered at 350 nm (Figure 2), ascribable to the combination of π→π* transitions of organic ligands and intramolecular charge transfer (ICT) involving the tetranuclear units [18,20,21]. As shown in Figure 3a, upon gradual addition of **D1** to **H1** solution in CHCl_3_, the intensity of the maximum absorption at 350 nm increases gradually along with the appearance of a shoulder centered at 370 nm. The changes observed in the absorption spectra during the titration indicated the encapsulation of **D1** guest molecules within the **H1**.

A plot of absorbance at 350 nm vs. **D1** equivalents (Figure 3b) exhibits a characteristic sigmoidal kinetic profile typically observed in the binding of substrates by enzymes or receptors containing multiple binding sites [20,32]. At low guest concentrations, the **D1** molecules added bind strongly to the **H1**, causing an initially flat and then sudden increase of the absorbance intensity. As the **D1** equivalents continue to increase, binding of **D1** guest to **H1** appears to become weaker, leading to a deviation of the titration curve from the initial tangent and a much more slow and gradual increase of the absorbance until reaching a saturation plateau. The capability of **D1** binding with **H1** can be estimated to be ~8 equivalents according to the intersection point of the initial tangent and the asymptote.

Notably, a plot of the absorbance at 370 nm vs [**D1**]/[**H1**] ratio displays a rare profile of well-defined multiple incremental steps, indicating sequential and cooperative host–guest interactions (Figure 3c). The stepwise increases in absorbance are observed in the [**D1**]/[**H1**] ranges of 0–2, 2–3, 3–8, and 8–23, respectively, which is ascribable to the stepwise encapsulation of **D1** guest molecules within different binding domains of the **H1** host, including the *endo*-cavity, *exo*-cavities, and the external pockets.

The binding behavior of the **D2** guest with **H1** was similarly investigated by the UV-Vis titration (Figure 3d–f). Similar sigmoidal kinetic profile was observed via a plot of absorbance at 350 nm vs. [**D2**]/[**H1**], but with a faster increase in the absorbance as the **D2** concentration increases at lower guest loadings. In the meanwhile, only three incremental steps in the [**D2**]/[**H1**] ranges of 0–2, 2–8, and 8–23 were observed. It is plausible that the smaller size of guest molecule (for example, the **D2** molecule bears a shorter side chain relative to **D1**) is beneficial to enhance the binding kinetic of guest encapsulation.

**Figure 4:**
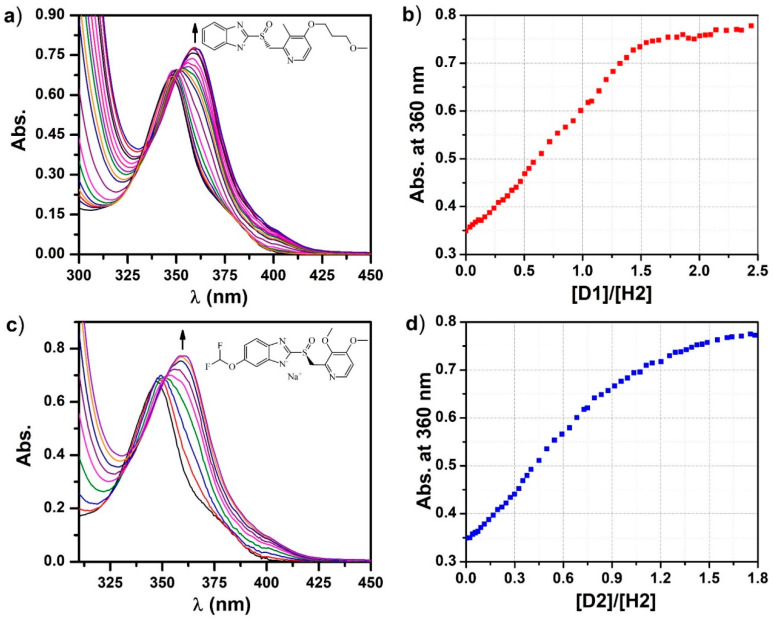
UV-Vis spectra of host **H2** upon titration with (**a**) **D1** and (**c**) **D2**, and plots of (**b**) **D1**/**H2** and (**d**) **D2**/**H2** molar ratio vs. absorbance at 360 nm based on the titration experiment.

In order to understand how the guest molecules bind to the **H1** host, control experiments employing the tetranuclear complex **H2** as the host were carried out under similar UV-Vis titration conditions (Figure 4). The binding of the **D1** guest molecules with **H2** is confirmed by the red shift of the **H2** absorption maxima from 347 nm to 360 nm upon gradual increase of the guest equivalents. The binding stoichiometry of **D1** or **D2** with **H2** is estimated to be ~1 as evidenced by the plots of absorbance vs. guest equivalents. This suggests that each *exo* cavity of the **H1** host tends to accommodate one molecule of the **D1** or **D2** guest. Therefore, it is plausible that the **H1** encapsulates six molecules of **D1** or **D2** through its six *exo* cavities, and two molecules of **D1** or **D2** within its *endo* cavity, while the remaining guest molecules are likely aggregating around the external triangular pockets.

**Figure 5:**
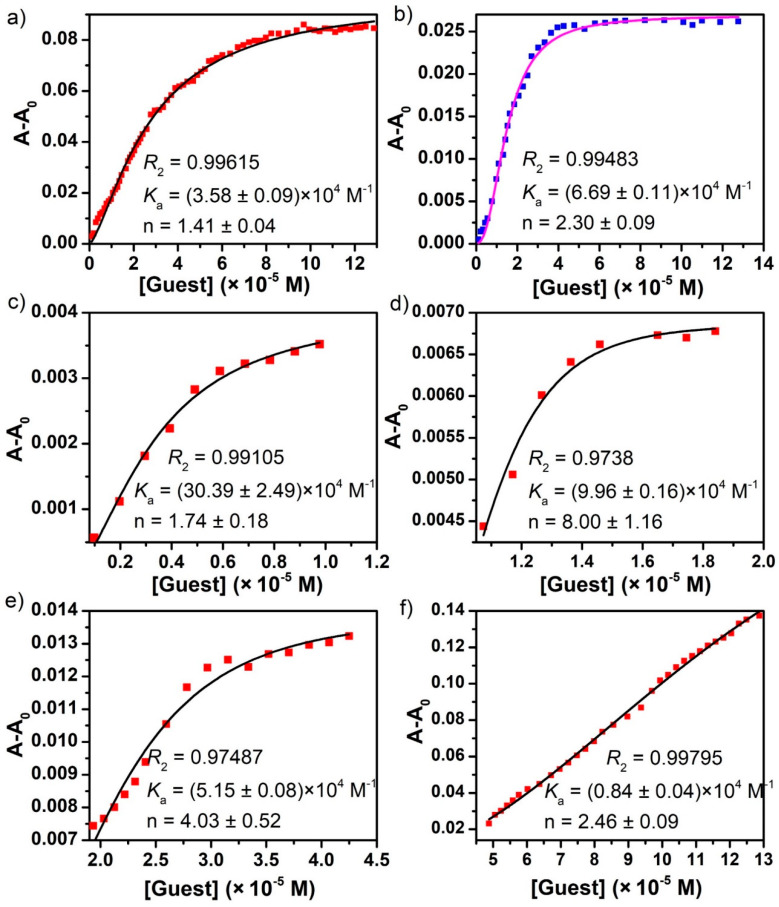
The nonlinear fits to Hill equation of UV-Vis titration experiments based on the absorption band centered at 350 nm for (**a**) **D1** ≅ **H1** and (**b**) **D2** ≅ **H1**, and at 370 nm in the [**D1**]/[**H1**] ranges of (**c**) 0−2, (**d**) 2−3, (**e**) 3−8, and (**f**) 8−23.

**Figure 6:**
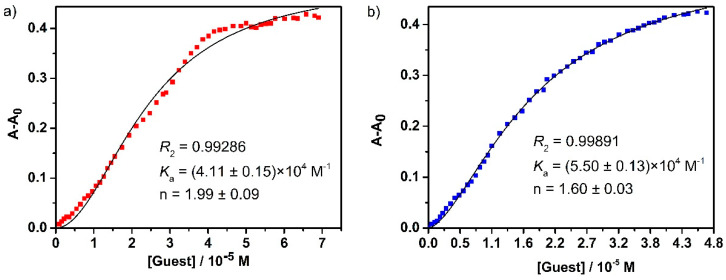
The nonlinear fits to Hill equation of UV-Vis titration experiments based on the absorption band centered at 360 nm for (**a**) **D1** ≅ **H2** and (**b**) **D2** ≅ **H2**.

Finally, the UV-Vis titration data was further analyzed using the well-known Hill equation^33^ in order to quantitatively understand the guest binding cooperativity. The binding association constants and the Hill coefficient values based on the nonlinear fit are listed in Table 1. The Hill equation is widely utilized for estimating the degree of cooperativity of the guest(s) binding to the receptor. The value of Hill coefficient provides a means to quantify the extent of cooperativity among multiple ligand binding stites. A Hill coefficient of one suggests independent binding, while the value different from one indicates multiple ligand binding corresponding to negatively (n < 1) or positively (n > 1) cooperative binding. As shown in Figure 5, the overall apparent association constants of guest molecules binding with **H1** were calculated to be 3.58 × 10^4^ M^−1^ and 6.69 × 10^4^ M^−1^ for **D1** and **D2**, respectively, compared with the corresponding values (4.11 × 10^4^ M^−1^ for **D1** and 5.55 × 10^4^ M^−1^ for **D2**) observed in the tetranuclear host **H2** (Figure 6). Taking into account the given Hill coefficient value (n > 1), the results suggested the positively cooperative and relatively strong overall binding between the host and guest [20,23].

It is worth noting that the stepwise guest encapsulation process revealed remarkable differences in the binding affinity of individual steps. The strongest binding affinity (i.e., 3.04 × 10^5^ M^−1^ for **D1** or 2.81 × 10^5^ M^−1^ for **D2**) was established in the first stage of the guest binding process involving two molecules of the guest, attributed to the encapsulation of the guest molecules inside the circumscribed *endo* cavity of **H1** through π···π interaction and hydrogen bonding between the host and guest molecules. A medium association constant found in the range of 5.15 × 10^4^–9.60 × 10^4^ M^−1^ in the following stage clearly suggested the entrapment of six molecules of guest by the six open, bowl-shaped *exo* cavites of **H1** through hydrogen bonding and hydrophobic interaction. Additional binding of the guest by the external pockets was supported by the weakest binding constant (i.e., 0.84 × 10^4^ M^−1^ for **D1** or 1.69 × 10^4^ M^−1^ for **D2**); however, the exact binding stoichiometry could not be determined, likely due to the fast exchange with the free guests.

## 3. Materials and Methods

### 3.1. General Information

Starting materials and solvents were obtained from commercial suppliers (Fisher Scientific, TCI, Alfa Aesar, etc.) and used without further purification. The metal-organic supercontainer **H1** [18] and a related reference compound, known as tetranuclear complex **H2** [30], were synthesized as described in the literature. The host materials were dried on a Schlenk line under vacuum at 120 °C for 4 h. UV-Vis absorption spectra were collected on a Perkin–Elmer Lambda 35 UV-Vis spectrophotometer at room temperature.

### 3.2. Solution UV-Vis Titration Experiments

Stock solutions of the hosts (**H1** and **H2**), called the titrand, were prepared in CHCl_3_ at a concentration of ~5 × 10^−6^ M. 25.00 mL of the host’s stock solution was then used to dissolve an accurately known mass of guest molecule, (*R*)-(+)-rabeprazole sodium (**D1**) or (*S*)-(−)-pantoprazole sodium (**D2**), called the titrant, wherein the guest concentration was 50~100 times greater than that of the host. 2.00 mL of the host solution (the titrand) was placed in a 10.0 mm quartz cell, upon which 0.01 to 2 mL of the titrant was added gradually using a syringe. After each addition, the cell was stoppered and inverted, and the UV-Vis spectrum was collected (at 25 °C) after 3 min to ensure complete mixing and reaching equilibration.

### 3.3. Calculation of Binding Constants from UV-Vis Titration Data

In order to evaluate the overall binding strength, the titration results were fitted to the nonlinear form of Hill equation [33,34].

$$\alpha =\frac{\Delta A}{{\Delta A}_{max}}=\frac{K_{a}{\left[L\right]}_{0}{}^{n}}{1+K_{a}{\left[L\right]}_{0}{}^{n}}=\frac{{\left[L\right]}_{0}{}^{n}}{\left(1/K_{a}\right)+{\left[L\right]}_{0}{}^{n}}$$

where ΔA (= A_obs_ − A_0_) is the change in absorbance, ΔA*_max_* is the maximum change of absorbance, [*L*]_0_ is initial guest concentration, *n* is the Hill coefficient, and *K_a_* is the association constant. A plot of ΔA against [*L*]_0_ can be used to estimate ΔA*_max_* and *K_a_*. The titration data were fit to this model using the nonlinear regression method within the Origin 9 software.

## 4. Conclusions

The guest binding behaviour of metal-organic supercontainer **H1** with two drug molecules, **D1** and **D2**, was investigated in chloroform solution using the UV-Vis titration technique. The results revealed highly intriguing cooperative and stepwise binding of the drug molecules with the multiple binding domains of **H1**. Taking into account the control experiments with the **H1** replaced by the tetranuclear complex **H2**, which represents the *exo* cavities of **H1**, it is suggested that the guest molecules were likely encapsulated sequentially by the different cavities of **H1**: two equivalents of the guest molecule were encapsulated inside the *endo* cavity of **H1**, followed by the subsequent entrapment of six molecules of the guest in the six *exo* cavities of **H1**, and finally, immobilization of additional guest molecules by the external pockets of **H1** was evidenced. The present study affords a thorough understanding of the cooperative binding behavior of metal-organic supercontainers featuring multiple binding domains, thus facilitating their potential biological applications such as drug delivery. We are currently addressing exciting opportunities along these lines.

## Figures and Tables

**Table 1:** Association constant and Hill coefficient determined by the nonlinear fit of the titration data to Hill equation.

	Association Constant (*K*_a_, × 10^4^ M^−1^)	Hill Coefficient (n)
**D1** ≅ **H1**	3.58 ± 0.09	1.41 ± 0.04
**D1** ≅ **H1***_endo_*	30.39 ± 2.49	1.74 ± 0.18
**D1** ≅ **H1***_exo_*	9.96 ± 0.16; 5.15 ± 0.08	8.00 ± 1.16; 4.03 ± 0.52
**D1** ≅ **H1***_external_*	0.84 ± 0.04	2.46 ± 0.09
**D2** ≅ **H1**	6.69 ± 0.11	2.30 ± 0.09
**D2** ≅ **H1***_endo_*	28.08 ± 5.84	1.43 ± 0.28
**D2** ≅ **H1***_exo_*	5.90 ± 0.14	3.90 ± 0.41
**D2** ≅ **H1***_external_*	1.69 ± 0.04	3.61 ± 0.59
**D1** ≅ **H2**	4.11 ± 0.15	1.99 ± 0.09
**D2** ≅ **H2**	5.55 ± 0.13	1.60 ± 0.03
